# Letter from Augusta

**Published:** 1885-02

**Authors:** J. M. Hull

**Affiliations:** Augusta, Ga.


### LETTER FROM AUGUSTA.

### HYDROCHLORATE OF COCAINE.

*Editors Atlanta Medical and Surgical Journal:*

GENTLEMEN—Having had several cases of apparent failure from the use of hydrochlorate of cocaine as an anæsthetic, I beg that you will allow space in your valuable journal for a short appendix to my article which appeared in January.

My surprise was great in my first experience of failure, as it was for the removal of a foreign body in the eye of a patient, for whom five days previously I had performed the same operation after instilling two drops of a two per cent. solution with perfect anæsthesia.

My second case was in the operation of iridectomy. I had to use eighteen drops of a four per cent. solution, two drops every three minutes, before I procured sufficient insensibility to operate. In three other cases I have had to repeat the application many times before obtaining sufficient anæsthesia to permit of operative procedure.

Now, in every one of these cases I had used a solution that I had had from five to eight days, and as the same solution had produced perfect anæsthesia for the same operations, and in two instances in the same patients, I concluded that the drug lost its strength by keeping in solution.

In order to assure myself if this were not true, I obtained a fresh solution and used it on the same patients in whom I had the failures, and found in every instance that anæsthesia was produced in one or two applications at most. My experience is that the drug does not keep for more than five or six days its full strength. I have now used it in over thirty cases with perfect results, except in the above mentioned cases. Very truly yours,

J. M. HULL, M. D.

Augusta, Ga., January, 1885.

